# Accurately Measuring the Infrared Spectral Emissivity of Inconel 601, Inconel 625, and Inconel 718 Alloys during the Oxidation Process

**DOI:** 10.3390/s24185906

**Published:** 2024-09-11

**Authors:** Longfei Li, Fayu Wang, Jiaying Gao, Kun Yu, Lan Wang, Yufang Liu

**Affiliations:** Henan Key Laboratory of Infrared Spectrum Measures and Applications, School of Physics, Henan Normal University, Xinxiang 453007, Chinayukun@htu.edu.cn (K.Y.);

**Keywords:** infrared spectral emissivity, measurement accuracy, nickel-based alloys, surface temperature correction, low thermal conductivity, thermal oxidation

## Abstract

Accurate measurement of the infrared spectral emissivity of nickel-based alloys is significant for applications in aerospace. The low thermal conductivity of these alloys limits the accuracy of direct emissivity measurement, especially during the oxidation process. To improve measurement accuracy, a surface temperature correction method based on two thermocouples was proposed to eliminate the effect of thermal conductivity changes on emissivity measurement. By using this method, the infrared spectral emissivity of Inconel 601, Inconel 625, and Inconel 718 alloys was accurately measured during the oxidation process, with a temperature range of 673–873 K, a wavelength range of 3–20 μm, and a zenith angle range of 0–80°. The results show that the emissivity of the three alloys is similar in value and variation law; the emissivity of Inconel 718 is slightly less than that of Inconel 601 and Inconel 625; and the spectral emissivity of the three alloys strongly increases in the first hour, whereafter it grows gradually with the increase in oxidation time. Finally, Inconel 601 has a lower emissivity growth rate, which illustrates that it possesses stronger oxidation resistance and thermal stability. The maximum relative uncertainty of the emissivity measurement of the three alloys does not exceed 2.6%, except for the atmospheric absorption wavebands.

## 1. Introduction

Nickel-based superalloys like Inconel 601, Inconel 625, and Inconel 718 have been widely used in aerospace, energy industry, and additive manufacturing for their high strength, creep resistance, and oxidative resistance at high temperatures up to 973 K [1,2]. Instrument components made by nickel-based superalloys, such as engine turbine blades, gas nozzles, and critical rotating parts, would be slowly oxidized under high temperatures, even if the surface of the alloy has been coated with anti-oxidation films [3]. The oxidation of the alloy will lead to dramatic changes in its spectral emissivity, which seriously affect the precision of radiation thermometry and heat transfer calculation [4,5,6]. The reported researches show that the oxidation kinetics of alloys is intricate, and the emissivity trend varies widely for different types of alloys with different compositions [7,8,9]. Therefore, it is important to accurately measure the spectral emissivity of nickel-based superalloys during the oxidation process.

In recent years, the emissivity of multiple nickel-based alloys has been measured under high temperatures with the increasing demand for accurate data of radiation properties. Kong et al. measured the spectral emissivity of oxidized Inconel 718 with a similar oxidation degree to engineering applications in the range of 2–15 μm and unveiled the mechanism of oxidation-induced emissivity variations by in-depth structural and compositional analysis [10]. The emissivity of oxidized Inconel 625, which is used currently for manufacturing solar receivers, was taken as a reference by Charpentier et al. to find new materials with a good creep resistance above 1400 K [11]. The following year, they measured the total hemispherical emissivity of various nickel-based alloys and finally confirmed that the Haynes 230 alloy was more suitable to build solar receivers via emissivity data comparison [12]. The emissivity of three Ni-based alloys was experimentally measured during oxidation by Li, et al. in near-infrared wavebands, and the observed oscillations of emissivity directly proved the major influence of thermal oxidation on spectral emissivity [13].

By carefully studying the measurement uncertainty of emissivity in the above works and the researches in terms of measuring spectral emissivity by the direct method [14], it is easy to find that the accuracy of surface temperature is the main factor to affect the emissivity measurement uncertainty. In order to accurately measure emissivity, researchers have proposed numerous experimental schemes to enhance the accuracy of target surface temperature measurement without compromising the radiation in the detection area. A commonly used method is to insert a thermocouple in a position close to the surface of sample, thereby reducing the influence of the temperature gradient between the thermocouple and the sample surface on the emissivity measurement [15,16,17,18]. To obtain the true temperature directly, the surface temperature calculation method based on the heat balance equation was proposed [19], and the contributions of heat conduction and heat convection were taken into account when the measurement was performed in air [20]. Moreover, the technique of infrared thermal imaging was introduced to correct the difference between the temperature of the sample center and the equivalent temperature of the total measurement area [21,22].

The majority of accurate emissivity measurements rely on a good heat conductivity of sample. Once the heat conductivity decreases, the deviation in the surface temperature will definitely increase, which would cause a large growth in emissivity measurement uncertainty. Compared with high thermal conductivity materials such as copper and aluminum, nickel-based alloys have poor thermal conductivity [23,24], and their surface temperature needs to be further corrected carefully when measuring their emissivity. In particular, the changes in thermal stress and oxygen content in the thermal oxidation process would lead to a disordered variation in sample heat conductivity [25], which will add a huge layer of difficulty for the surface temperature calculation. Therefore, it is significant to explore a new surface temperature calculation method, which could eliminate the effect of heat conductivity changes on the spectral emissivity measurement in air.

In this paper, a method accurately monitoring the surface temperature of low thermal conductivity alloys during oxidation was presented. Two thermocouples were set on the side of the sample. The thermal conductivity and initial surface temperature of the sample were corrected in real time by the measured values of the two thermocouples, and the accurate surface temperature was obtained by an iterative calculation of emissivity and surface temperature. By using this method, the directional spectral emissivity of three kinds of alloys (Inconel 601, Inconel 625, and Inconel 718) was accurately measured at 673 K, 773 K, and 873 K, and the effects of temperature, wavelength, zenith angle, and oxidation on spectral emissivity were investigated. This work can provide a new surface temperature calculation method for the spectral emissivity measurement of low thermal conductivity materials, and it can enrich the spectral emissivity data of nickel-based alloys, which would provide support for radiation temperature measurement and heat transfer calculation in aerospace and thermal management.

## 2. Surface Temperature Calculation Method

**Figure 1:**
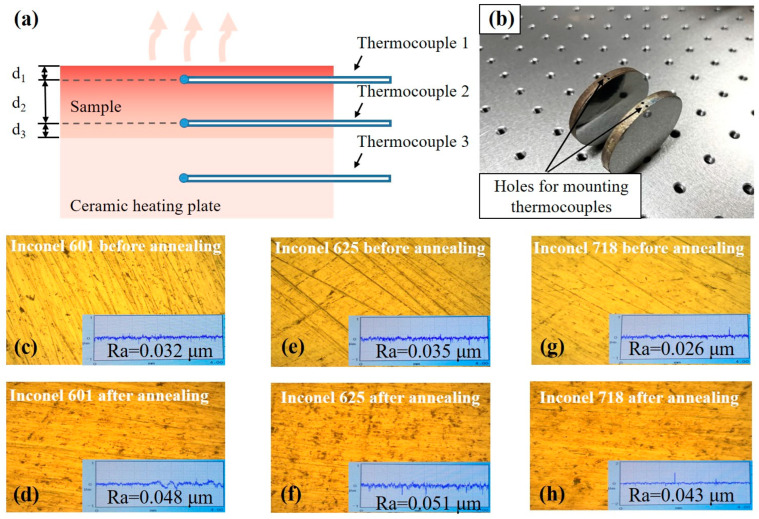
(**a**) Diagram of surface temperature measurement for nickel-based alloys; (**b**) Prepared samples with two thermocouple holes; (**c**–**h**) The roughness and metallography measured before and after the annealing process of Inconel 601, Inconel 625, and Inconel 718, respectively.

In order to accurately obtain the surface temperature of a low thermal conductivity sample at a high temperature, as shown in Figure 1, two small holes are drilled on the side of the sample, and two K-type thermocouples undergoing the same calibration process are set in the two holes. The distance between Thermocouple 1 and the sample surface is marked as *d*_1_, the distance between Thermocouple 1 and Thermocouple 2 is *d*_2_, and the distance between Thermocouple 2 and the back of the sample is *d*_3_. In addition, Thermocouple 3 is arranged in the ceramic heating plate for temperature control of the sample. In the emissivity measurement process, when the sample reaches thermal equilibrium, the edge effect can be ignored, and the heat transfer along the central axis of sample is similar to one-dimensional heat transfer. This means that the heat flow from Thermocouple 2 to Thermocouple 1 is equal to the heat flow from Thermocouple 1 to the sample surface.

(1) $${q^{\prime \prime }}_{in,Conduction}=\frac{k_{s}}{d_{2}}(T_{2}-T_{1})=\frac{k_{s}}{d_{1}}(T_{1}-T_{S})$$

where *T*_1_ and *T*_2_ are the measured values of Thermocouple 1 and Thermocouple 2, respectively, *T*_S_ is the sample surface temperature, and *k*_S_ is the thermal conductivity of sample.

(2) $$T_{S}=\left(1+\frac{d_{1}}{d_{2}}\right)T_{1}-\frac{d_{1}}{d_{2}}T_{2}$$

According to Equation (2), the sample surface temperature *T*_S_ can be directly calculated by the measured *T*_1_ and *T*_2_. However, this calculated value cannot be directly used as the final surface temperature because *d*_1_ and *d*_2_ are often imprecise due to the machining position deviation of the thermocouple hole.

When the sample reaches thermal equilibrium, the heat flow transferred from the sample surface to the surrounding environment can be expressed as follows:

(3) $${q^{\prime \prime }}_{out,Radiation}=\varepsilon_{\cap }\sigma \left(T_{S}^{4}-T_{sur}^{4}\right)+\frac{3k_{b}}{4l}Nu\left(T_{S}-T_{b}\right)$$

where $\varepsilon_{\cap }$ is the hemisphere-total emissivity of the sample and obtained by integrating spectral emissivity, which can be calculated by the initial surface temperature [26]. σ is the Stefan–Boltzmann constant, $T_{\mathrm{sur}}$ is the temperature of the surrounding environment, Nu is the Nusselt number, $k_{b}$ and $T_{b}$ represent the mean thermal conductivity and the temperature of the boundary layer around the sample, and *l* represents the characteristic length of heat exchange. The heat balance equation can be expressed as follows.

(4) $$\varepsilon_{\cap }\sigma \left(\left(T_{S}^{4}-T_{sur}^{4}\right)\right)+\frac{3k_{b}}{4l}(Nu\left(T_{S})-T_{b}\right)-\frac{k_{s}}{d_{1}}(T_{1}-T_{S})=0$$

**Figure 2:**
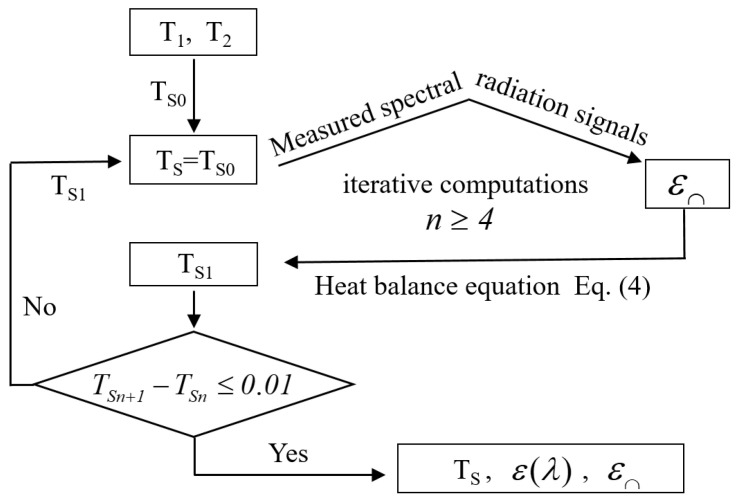
Schematic diagram of the iterative computation.

As shown in Figure 2, the temperature *T*_S0_ calculated by Equation (2) is taken as the initial temperature firstly. Then, the directional spectral emissivity of the sample can be calculated via the measured spectral radiation signals, and the hemispherical emissivity is obtained by spectrum and space integrals. The obtained hemispherical emissivity is substituted into Equation (4) to further calculate the surface temperature *T*_S1_. If the difference between the obtained surface temperature and the initial temperature exceeds 0.1 K, *T*_S1_ will be substituted into the emissivity calculation formula again. Then, the second generation hemispherical emissivity is calculated, and the second generation temperature *T*_S2_ can be worked out. The iterative computation will be successively performed until the difference between the two continuously calculated surface temperatures is no more than 0.1 K. Finally, the surface temperature and the emissivity of sample can be accurately solved.

## 3. Samples

Three kinds of cylindrical nickel-based alloy samples (Inconel 718, Inconel 625, and Inconel 601, with a diameter of 50 mm and a thickness of 5 mm, provided by Shenzhen Qiyi Metal Materials Co., Ltd., Shenzhen, China) were prepared by the same turning process to have almost identical surface topography. The corresponding compositions shown in Table 1 were provided by the supplier above. Two thermocouple holes with a depth of 25 mm and a diameter of 1 mm were punched on the side of each sample.

Each sample was polished by a metallographic polishing machine (EcoMet30) with 80 grit, 120 grit, and 180 grit sandpaper successively. In order to ensure the uniformity of the polished surface, the sample was rotated 90° when switching the sandpaper each time, and then it was polished along the direction perpendicular to the surface textures obtained from the last polishing. The polishing was finished until the traces coming from the last polishing were completely removed. The polished surfaces were washed with acetone and anhydrous ethanol to remove the oil and impurities remaining on the surface. The polished samples were cleaned by distilled water and dried by an air blower. Then, the surface roughness and the metallography of each sample were measured by a roughness tester (TIME 3221) and a metalloscope (Motic: BA310Met). Finally, the prepared samples were annealed at 873 K for 3 h in a vacuum muffle furnace to eliminate the influence of surface stress on the thermal radiation. After the annealing process, the roughness and the metallography were measured again, and the results are shown in Figure 1.

## 4. Device and Method for Measuring Spectral Emissivity

**Figure 3:**
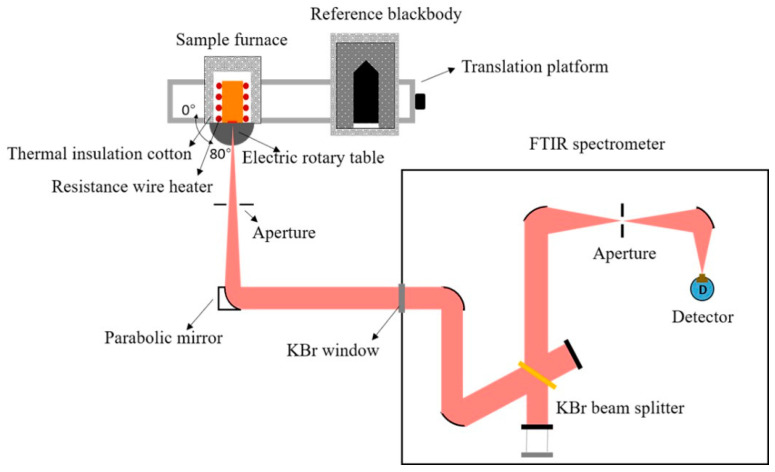
Schematic diagram of the directional spectral emissivity measurement device.

The device for measuring the spectral emissivity in air is shown in Figure 3 and mainly includes a sample furnace, a reference blackbody (SOTECH R970), a removable shutter, an aperture, a parabolic mirror (f = 406 mm), and a Fourier transform infrared (FTIR) spectrometer (Bruker, Karlsruhe, Germany, Vertex 70 V) with a KBr beam splitter and a DTGLaS detector. Samples can be fixed in the front of sample furnace and heated by an alumina ceramics heating plate, which is firmly fixed on a self-made resistance wire (Kanthal, Sandviken, Sweden, A-1/APM) heater. An S-type thermocouple (above mentioned as Thermocouple 3) embedded in the alumina ceramics heating plate is used to monitor the temperature of the heating plate and to provide feedback signals to a PID thermostat (SHIMADEN, Tokyo, Japan, SR23) with the aim of controlling the temperature of the heating plate to be stable near the set value. The maximum heating temperature is up to 1000 K, which mainly determined by the thickness and the thermal conductivity of the samples. In this paper, the nickel-based alloy samples with a thickness of 5 mm can only be heated up to 881 K according to the preliminary test. Samples can be rotated from the normal direction (0°) to 80° by an electric rotary table (RAK100G). Thermal radiation from the sample or the reference blackbody, selected by a displacement platform (PSA300-11-X), is reflected by the parabolic mirror into the FTIR spectrometer and converted into a recordable electrical signal. The shutter is self-made and used to calibrate the response of the radiation detection system. Finally, spectral emissivity in the wavelength range of 3–20 μm can be calculated based on the measured radiation signals of the sample, the blackbody, and the shutter by carefully considering the surface temperature correction of the sample. 

When measuring the sample, the signal ${\tilde{L}}_{S}$ can be expressed as:

(5) $${\tilde{L}}_{s}=s\cdot \left\{\left.\varepsilon_{s}(T_{s})\cdot L_{Planck}(T_{s})+\left[1-\varepsilon_{s}(T_{s})\right]\cdot \varepsilon_{\mathrm{sur}}\cdot L_{Planck}(T_{sur})+L_{Background}\right\}\right..$$

where *s* is the spectral response of the device, $L_{Planck}(T_{S})$ and $L_{Planck}(T_{\mathrm{sur}})$ represent spectral radiance of the sample and the surrounding environment, respectively, $\varepsilon_{s}(T_{s})$ is the spectral emissivity of the sample, $\varepsilon_{\mathrm{sur}}$ is the spectral emissivity of the environment, and $L_{Background}$ is the spectral background radiation of the FTIR spectrometer.

When measuring the blackbody, the directional spectral emissivity of the reference blackbody $\varepsilon_{BB}$ can be approximated to 1, and the measured signal ${\tilde{L}}_{BB}$ can be expressed as follows:

(6) $${\tilde{L}}_{BB}=s\cdot \left[\varepsilon_{BB}L_{Planck}(T_{BB})+L_{Background}\right].$$

The directional spectral emissivity of the sample can be calculated by the ratio of the sample signal to the blackbody signal as follows:

(7) $$\varepsilon_{s}(T_{s})=\frac{{\tilde{L}}_{s}}{{\tilde{L}}_{BB}}\cdot \frac{\varepsilon_{BB}L_{Planck}(T_{BB})+L_{Background}}{L_{Planck}(T_{s})-\varepsilon_{\mathrm{sur}}\cdot L_{Planck}(T_{sur})}-\frac{\varepsilon_{\mathrm{sur}}\cdot L_{Planck}(T_{sur})+L_{Background}}{L_{Planck}(T_{s})-\varepsilon_{\mathrm{sur}}\cdot L_{Planck}(T_{sur})},$$

where $\varepsilon_{\mathrm{sur}}\cdot L_{Planck}(T_{sur})$ is calculated by controlling the environment around the sample, and $L_{Background}$ is calibrated by using the multi-temperature method.

## 5. Measurement Procedure

Two thermocouples were embedded in an annealed sample directly, and then the sample was installed on the heating plate by two fastening screws. The sample was heated to 673 K, 773 K, and 873 K, respectively. After the sample reached thermal equilibrium, the radiation signal of the blackbody at different temperatures and that of the shutter at room temperature were measured first. Then, the sample furnace was moved to the measurement position, and the radiation signal of sample was measured in 0–80° with a zenith angle interval of 10°. Meanwhile, the values of Thermocouple 1 and Thermocouple 2 were recorded during each measurement. Finally, air was slowly blown to the sample surface, and the sample was uniformly oxidized for 12 h with a measurement time interval of 2 h. Each measurement was repeated three times to reduce the measurement error. After each set of measurements in 0–80° was completed, the spectral emissivity and surface temperature of the sample were obtained according to the iterative calculation method. When all measurements were completed, the sample was cooled to room temperature and stored in a vacuum chamber. Following the above steps, three types of samples (Inconel 601, Inconel 625, and Inconel 718) were measured sequentially.

## 6. Measurement Results and Analysis

### 6.1. Spectral Emissivity of Inconel 601, Inconel 625, and Inconel 718 at Different Temperatures, Wavelengths, and Zenith Angles

**Figure 4:**
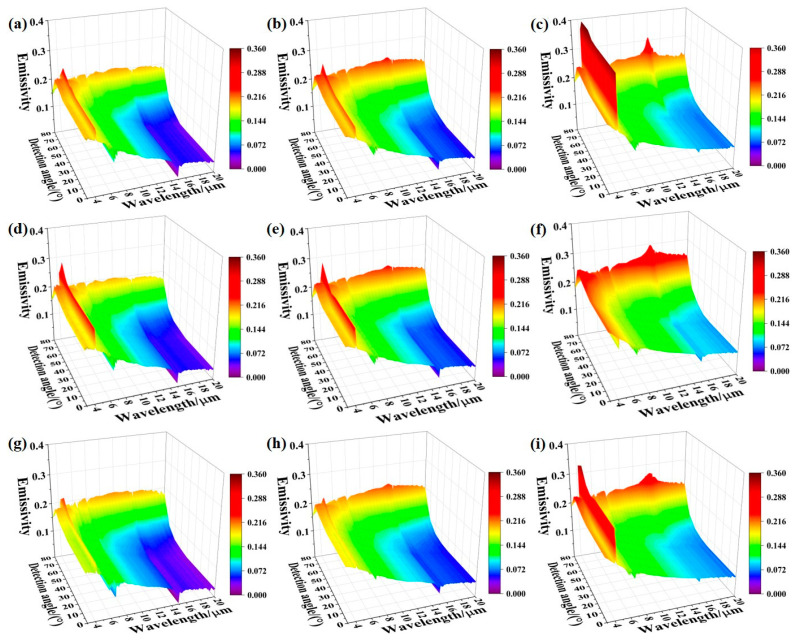
Infrared spectral emissivity of the three kinds of nickel-based alloys measured in 0–80° and at different temperatures: (**a**–**c**) Inconel 601 at 673 K, 773 K, 873 K, respectively; (**d**–**f**) Inconel 625 at 673 K, 773 K, 873 K, respectively; (**g**–**i**) Inconel 718 at 673 K, 773 K, 873 K, respectively.

Figure 4 shows the first set of spectral emissivity data in 0–80° of the three nickel-based alloys (Inconel 601, Inconel 625, Inconel 718), which was measured when the sample had just reached thermal equilibrium. It can be found that the emissivity of the three kinds of alloys is similar in value and variation law, and it decreases with the increase in wavelength under small zenith angles. This is consistent with the characteristics of metal emissivity [27]. When the zenith angle exceeds 70°, the emissivity increases slowly with the increase in wavelength. The difference between the emissivity value at a short wavelength and that at a long wavelength gradually decreases with the increase in temperature. The possible reason is that the sample in the heating process is not protected against oxidation, which leads to the formation of a slight oxide layer on the sample surface. The phenomenon is more obvious at 873 K for the thickness of oxide layer increases with the heating temperature. In addition, the spectral emissivity oscillates violently at 4.3 μm, 5–8 μm, and 15 μm, and these oscillations are mainly caused by the absorption of air (water and carbon dioxide) in the optical path before the radiation enters the FTIR spectrometer.

### 6.2. Effect of Wavelength on the Emissivity of Inconel 601, Inconel 625, and Inconel 718

**Figure 5:**
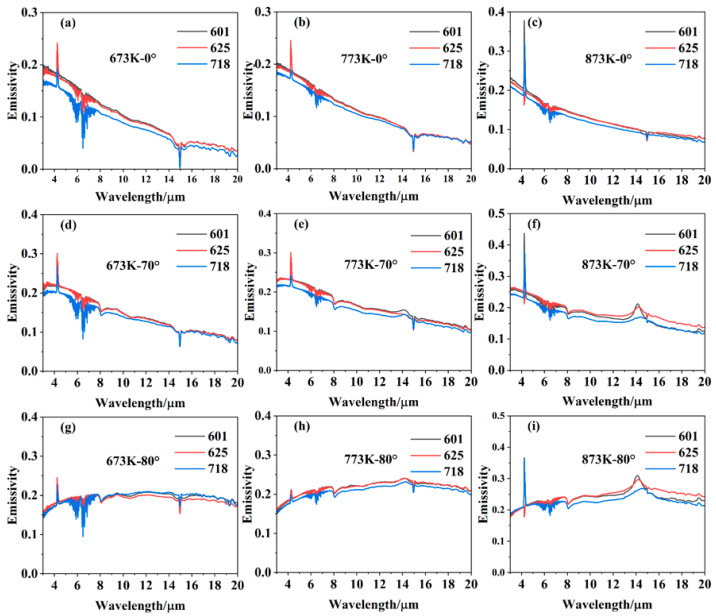
Spectral emissivity as a function of wavelength for the three kinds of nickel-based alloys (Inconel 601, Inconel 625, and Inconel 718) under different temperatures and measuring zenith angles: (**a**–**c**) measured in the normal direction and at 673 K, 773 K, 873 K; (**d**–**f**) measured at 70° and at 673 K, 773 K, 873 K; (**g**–**i**) measured at 80° and at 673 K, 773 K, 873 K.

Comparing the data of the three kinds of alloys, as shown in Figure 5, it is obvious that the emissivity of Inconel 601 is close to that of Inconel 625, while they are slightly higher than that of Inconel 718. The phenomenon is noteworthy because the nickel content of 718 alloy is small, while the iron content is relatively large. According to the reported emissivity data of pure Ni and pure Fe, the emissivity of Inconel 718 should be higher than that of Inconel 601 and Inconel 625 from the perspective of material composition [28,29]. The reason for the small emissivity value of Inconel 718 should be that the hardness of Inconel 718 is high, and its surface roughness is less than that of Inconel 601, and Inconel 625 when polished with the same sandpaper as shown in Figure 1. Therefore, the reason for the emissivity difference would be mainly attributed to the different roughness of the polished surfaces. 

The emissivity of the three alloys increases with the increase in temperature, and the amplitude of the growth rate decreases with wavelength, which in consistent with the Hagen–Rubens relationship [30]. When the temperature increases, the vibration of metal ions becomes more intense, which will lead to the collision between electrons and metal ions becoming more frequent. The increase in temperature will result in a resistance increase, and this resistance increase further leads to the increase in emissivity finally. It is worth noting that the emissivity value of the Inconel 718 alloy is hard to be distinguished from that of Inconel 601 and Inconel 625 at 80°, which might be owing to the fact that the influence of the surface micro-grooves on the spectral emissivity is weakened at large zenith angles.

### 6.3. Effect of Zenith Angle on the Emissivity of Inconel 601, Inconel 625, and Inconel 718

**Figure 6:**
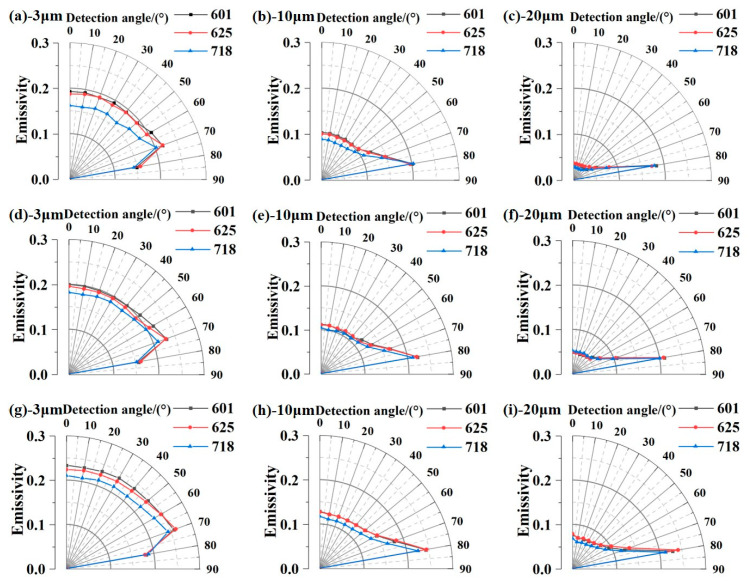
Spectral emissivity as a function of measuring zenith angle for the three kinds of nickel-based alloys (Inconel 601, Inconel 625, and Inconel 718) at (**a**–**c**) 673 K, (**d**–**f**) 773 K, and (**g**–**i**) 873 K, respectively.

In order to study the spatial distribution of the spectral emissivity of nickel-based alloys, the data of Inconel 601, Inconel 625, and Inconel 718 at three wavelengths (3 μm, 10 μm, and 20 μm) were selected and plotted in Figure 6. It can be found that the directional spectral emissivity of the three nickel-based alloys shows two different trends under short wavelengths and long wavelengths. At short wavelengths, the emissivity hardly changes in the zenith angle range of 0–50°, it increases slowly with a zenith angle of 50–70°, and finally it decreases rapidly with zenith angle once the measurement angle exceeds 70°. At long wavelengths, the emissivity increases slowly before 60° and rapidly increases with the zenith angle until 80°. The phenomenon was found to be consistent with the fundamental property of metals and can be explained by the changes in metal optical constants and the emissivity prediction models based on Fresnel’s formula [31].

### 6.4. Effect of Thermal Oxidation on the Spectral Emissivity of Inconel 601, Inconel 625, and Inconel 718

**Figure 7:**
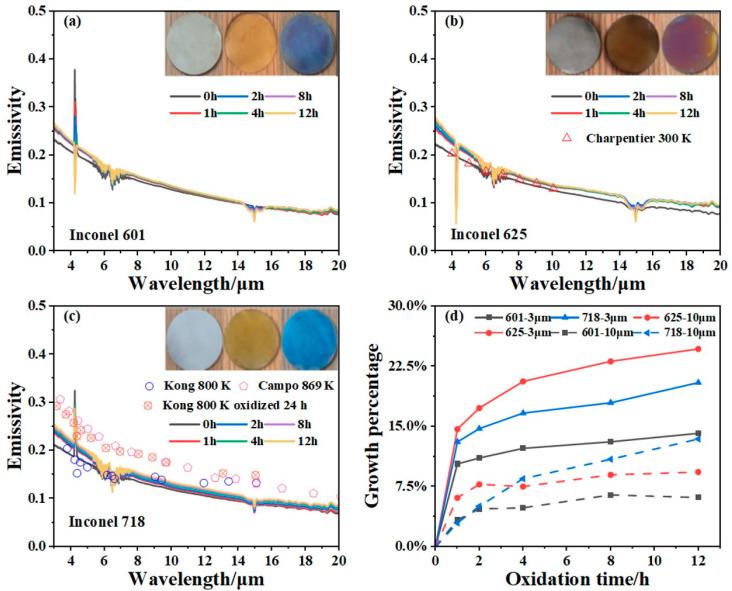
Spectral emissivity measured during the oxidation process for (**a**) Inconel 601; (**b**) Inconel 625; (**c**) Inconel 718. (**d**) Comparison of the emissivity growth rate of Inconel 601, Inconel 625, and Inconel 718 in 12 h and at 3 μm and 10 μm, respectively.

As shown in Figure 7, the surface color of the polished alloys is silver when the samples have not been oxidized. Keeping the surface temperature at 873 K, the surface colors of the three kinds of alloys rapidly turn to brown within 30 min, where the Inconel 625 alloy possesses the largest degree of discoloration. Then, the surface colors of these samples gradually turn blue with heating time, and the color of Inconel 625 finally turns purple in 12 h. If we focus on the change in emissivity, it can be found that the spectral emissivity of all samples has a strong increase in the first hour, and the growth rate of emissivity gradually slows down with the increase in oxidation time. In the process of continuous heating, the changes in the surface color and emissivity are related to the oxide film on the sample [32,33]. The accumulation of the thickness of the oxide film changes the interference effect on the alloy surface, which will lead to the sample surface having different colors in the visible waveband and different spectral emissivity in the infrared waveband. In addition, the literature data of the spectral emissivity of Inconel 625 and Inconel 718 are shown in Figure 7b,c to be compared with the measured data. The result of the oxide-free Inconel 625 reported by Charpentier et al. is in good agreement with the data measured when the sample just reached thermal equilibrium [11]. Moreover, the data of Inconel 718 measured by Kong et al. before oxidation are also in accordance with the results in this paper [10]. Because the sample of Kong et al. was oxidized for 24 h, much longer than the oxidation time in our experiments, the reported data show higher values than the measured result, which conforms well with the emissivity growth role with oxidation degree. It is worth noting that the emissivity data reported by Campo et al. before oxidation are much higher than the measured result, which could be attributed to the large roughness (Ra = 1.3 μm) of their brushed samples [7]. The consistency between the literature data and the measured results suitably illustrates the reliability of the proposed spectral emissivity measurement method for nickel-based alloys with low thermal conductivity.

Finally, comparing the spectral emissivity changes in the three kinds of alloys during the oxidation process, it can be concluded from Figure 7d that Inconel 601 has the lowest emissivity growth rate, which means that it has stronger oxidation resistance and stability, and would be more suitable for applications needing better oxidation resistance and precise temperature control. The good oxidation resistance of Inconel 601 may be attributed to the high content of aluminum, which will be rapidly oxidized in air, and a dense protective film that would be covered on the surface to prevent the further oxidation of the sample. 

### 6.5. Uncertainty Evaluation

The uncertainty of spectral emissivity measurement of the three kinds of alloys is determined by Equation (8),

(8) $$\begin{array}{l} \Delta \varepsilon^{2}={\left(\frac{\partial \varepsilon }{\partial T_{b}}\Delta T_{b}\right)}^{2}+{\left(\frac{\partial \varepsilon }{\partial T_{s}}\Delta T_{s}\right)}^{2}+{\left(\frac{\partial \varepsilon }{\partial T_{sur}}\Delta T_{sur}\right)}^{2}\\ +{\left(\frac{\partial \varepsilon }{\partial V_{b}}\Delta V_{b}\right)}^{2}+{\left(\frac{\partial \varepsilon }{\partial V_{s}}\Delta V_{s}\right)}^{2}+{\left(\frac{\partial \varepsilon }{\partial \varepsilon_{b}}\Delta \varepsilon_{b}\right)}^{2} \end{array},$$

where $\Delta T_{b}$, $\Delta T_{s}$, $\Delta T_{sur}$, $\Delta V_{b}$, $\Delta V_{s}$ and $\Delta \varepsilon_{b}$ are the uncertainties of the blackbody temperature, sample surface temperature, ambient temperature, measurement signal of blackbody radiation, measurement signal of sample radiation, and emissivity of blackbody, respectively. The combined uncertainty of sample temperature can be calculated using the following formula:

(9) $$\Delta T_{s}=\sqrt{\Delta c^{2}+\Delta s_{1}^{2}+\Delta s_{2}^{2}},$$

where $\Delta s_{1}$ and $\Delta s_{2}$ represent the temperature measurement uncertainty of Thermocouple 1 and Thermocouple 2, respectively, and Δc is the uncertainty introduced by the iterative calculation of surface temperature.

After the temperatures of the blackbody and the sample were sufficiently stable, the radiation signals were measured three times in each zenith angle. The mean value and uncertainty of the blackbody radiation signal or the sample radiation signal can be expressed as follows:

(10) $$\bar{S}=\frac{1}{n}\sum_{i=1}^{n}S_{i},$$

(11) $$\Delta S=\sqrt{\frac{\sum_{i=1}^{n}\left(S_{i}-\bar{S}\right)}{n\left(n-1\right)}}.$$

According to the above method, the normal spectral emissivity measurement uncertainty of Inconel 601, Inconel 625, and Inconel 718 was calculated at the temperatures of 673 K and 873 K and at the wavelengths of 3 μm and 10 μm, respectively. The data are listed in Table 2, Table 3 and Table 4.

By analyzing the uncertainty contribution, the measurement accuracy of the sample surface temperature is still the main factor affecting the measurement uncertainty of the spectral emissivity of nickel-based alloys, although the surface temperature has been corrected by two thermocouples. The maximum relative uncertainty of the three kinds of alloys does not exceed 2.6% except the atmospheric absorption wavebands, which can meet the data accuracy requirements for the majority of applications.

## 7. Conclusions

In this paper, the infrared spectral emissivity of Inconel 601, Inconel 625, and Inconel 718 alloys was measured by the direct measurement method to provide accurate emissivity data for applications in aerospace, energy industry, and additive manufacturing. A surface temperature correction method based on two thermocouples was proposed to eliminate the effect of low thermal conductivity and its changes on the direct spectral emissivity measurement of nickel-based alloys during the thermal oxidation process. The measurement results illustrate that the spectral emissivities of the three kinds of nickel-based alloys are similar with each other in terms of value and variation law, and the emissivity of Inconel 718 is slightly lower than that of Inconel 601 and Inconel 625. More important is that Inconel 601 can be proved to be further resistant to thermal oxidation under high temperature by comparing the emissivity during the oxidation process. This work may provide emissivity data for material selection and heat transfer calculations in aircraft design, as well as help to improve the accuracy of the radiation temperature measurement of nickel-based alloy components in industrial production.

## Figures and Tables

**Table 1:** Compositions of three kinds of nickel-based alloys.

Elements/%	Ni	Cr	Fe	Mo	Nb
Inconel 718	50.0–55.0	17.0–21.0	11.1–24.6	2.8–3.3	4.75–5.50
Inconel 625	54.9–68.9	20.0–23.0	Max.5.0	8.0–10.0	3.15–4.15
Inconel 601	58.0–63.0	21.0–25.0	7.7–20.0	/	/
**Elements/%**	**C**	**Mn**	**Si**	**S**	**Cu**
Inconel 718	Max.0.08	Max.0.35	Max.0.35	Max.0.015	Max.0.30
Inconel 625	Max.0.1	Max.0.5	Max.0.5	Max.0.015	/
Inconel 601	Max.0.1	Max.1.0	Max.0.5	Max.0.015	Max.1.0
**Elements/%**	**Al**	**Ti**	**B**	**Co**	**P**
Inconel 718	0.2–0.8	0.65–1.15	Max.0.006	Max.1.0	Max.0.015
Inconel 625	Max.0.4	Max.0.4	/	Max.1.0	Max.0.015
Inconel 601	1.0–1.7	/	/	/	/

**Table 2:** Uncertainty evaluations (%) of the normal spectral emissivity of the Inconel 601 alloy.

Parameters	T = 673 K		T = 873 K	
	λ = 3 μm ε = 0.187	λ = 10 μm ε = 0.099	λ = 3 μm ε = 0.239	λ = 10 μm ε = 0.134
$\Delta T_{b}$	0.058	0.030	0.054	0.029
$\Delta T_{s}$	0.217	0.102	0.547	0.125
$\Delta T_{sur}$	0.081	0.137	0.113	0.168
$\Delta V_{b}$	0.061	0.076	0.062	0.082
$\Delta V_{s}$	0.018	0.020	0.021	0.027
$\Delta \varepsilon_{b}$	0.003	0.003	0.003	0.003
Δε	0.246	0.189	0.565	0.227
Δε/ε	1.318	1.913	2.362	1.692

**Table 3:** Uncertainty evaluations (%) of the normal spectral emissivity of the Inconel 625 alloy.

Parameters	T = 673 K		T = 873 K	
	λ = 3 μm ε = 0.184	λ = 10 μm ε = 0.098	λ = 3 μm ε = 0.234	λ = 10 μm ε = 0.133
$\Delta T_{b}$	0.056	0.024	0.049	0.022
$\Delta T_{s}$	0.202	0.098	0.538	0.096
$\Delta T_{sur}$	0.082	0.145	0.096	0.146
$\Delta V_{b}$	0.060	0.076	0.063	0.085
$\Delta V_{s}$	0.013	0.018	0.018	0.022
$\Delta \varepsilon_{b}$	0.003	0.003	0.003	0.003
Δε	0.233	0.192	0.552	0.196
Δε/ε	1.266	1.962	2.360	1.470

**Table 4:** Uncertainty evaluations (%) of the normal spectral emissivity of the Inconel 718 alloy.

Parameters	T = 673 K		T = 873 K	
	λ = 3 μm ε = 0.163	λ = 10 μm ε = 0.087	λ = 3 μm ε = 0.218	λ = 10 μm ε = 0.122
$\Delta T_{b}$	0.064	0.031	0.049	0.027
$\Delta T_{s}$	0.218	0.098	0.548	0.213
$\Delta T_{sur}$	0.076	0.137	0.075	0.128
$\Delta V_{b}$	0.054	0.083	0.063	0.065
$\Delta V_{s}$	0.010	0.014	0.018	0.021
$\Delta \varepsilon_{b}$	0.003	0.003	0.004	0.004
Δε	0.246	0.190	0.559	0.258
Δε/ε	1.507	2.188	2.563	2.117

## Data Availability

The data presented in this study are available in this paper.
